# A comparative study of single *Theileria lestoquardi* and mixed infections with *Theileria ovis*

**DOI:** 10.1186/s13071-021-04864-6

**Published:** 2021-07-20

**Authors:** Salama Al-Hamidhi, Elshafie I. Elshafie, Saeed Yaghfoori, W. Ivan Morrison, Eugene H. Johnson, Hamza A. Babiker

**Affiliations:** 1 Department of Biochemistry, College of Medicine and Health Sciences, Sultan Qaboos University, AlKhoud 123, PO Box 35, Muscat, Oman; 2 Department of Animal and Veterinary Sciences, College of Agricultural and Marine Sciences, Sultan Qaboos University, Muscat, Oman; 3 Central Veterinary Research Laboratories, Al Amarat, P.O. Box 8067, Khartoum, Sudan; 4 Laboratory for Hormozgan Veterinary, Bandar Abbas, Iran; 5 Division of Infection and Immunity, The Roslin Institute, Royal (Dick) School of Veterinary Studies, University of Edinburgh, Edinburgh, UK; 6 Institute of Immunology and Infection Research, School of Biological Sciences, University of Edinburgh, Edinburgh, UK

**Keywords:** Theileriosis, *Theileria lestoquardi*, *T. ovis*, Genetic diversity

## Abstract

**Background:**

Epidemiological surveys in Oman have revealed a high prevalence of the co-occurrence of the pathogenic *Theileria lestoquardi* and the non-pathogenic *Theileria ovis* among sheep in the Barka region, Oman. Our most recent data illustrated an interaction and reduced mortality risk in animals co-infected with *T. lestoquardi* and *T. ovis*, suggesting that the latter confers protection against pathogenicity of *T. lestoquardi*. The present study extends the above findings and examines disease outcomes; clinical markers, hematological parameters, and parasite density in mixed and single *T. lestoquardi* infections.

**Methods:**

A total of 390 blood samples were collected from 16 sheep pens located in Barka, Oman between July and November 2019. *Theileria* spp. were detected and quantified using qPCR assay targeting *18S rRNA*, and the extent of genetic diversity was estimated by a panel of *T. lestoquardi* specific micro- and mini-satellites. The association of some disease markers with the presence of *Theileria* spp. and genetic diversity was tested.

**Results:**

*Theileria* spp. were detected in 75 (19.2%) sheep; of these 65 (86.7%) had mixed infections (*T. lestoquardi* plus *T. ovis*), 8 (10.6%) were infected with *T. lestoquardi* alone, and 2 (2.7%) with only *T. ovis*. Exotic breeds had a higher risk for *Theileria* spp. infection. The density (*18S rRNA* gene copies) of both parasites was higher in single infection against mixed infection, and there was a relatively lower density of *T. lestoquardi* in mixed infections. However, there was no difference in hematological indices between single *T. lestoquardi* and mixed infections. High genetic diversity was observed among *T. lestoquardi* in Barka, with no differences of *T. lestoquardi* in single and mixed infections. The extent of diversity seen in Barka was higher (*He* = 0.772) than that reported in Oman in 2019 (*He* = 0.582), with distinct *T. lestoquardi* genotypes.

**Conclusion:**

The lower density of *T. lestoquardi* as mixed infection with *T. ovis* compared to single infection supports the hypothesis that *T. ovis* confers protection against lethal *T. lestoquardi* infection. However, there were no differences in disease correlations (clinical markers, hematological parameters, and density of parasites) or the extent of diversity of *T. lestoquardi* between the two types of infection. The presence of distinct *T. lestoquardi* genotypes in Barka, compared to that reported earlier in Oman, likely reflects movement of carrier animals and highlights the need for further analysis of the parasite populations to inform novel approaches for controlling malignant ovine theileriosis.

**Graphical Abstract:**

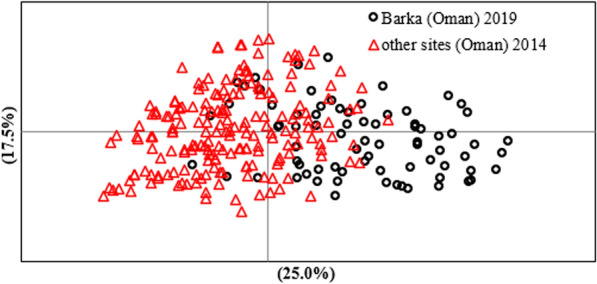

## Background

Malignant ovine theileriosis is an important hemoprotozoal disease of sheep and goats in tropical and subtropical regions caused by pathogenic *Theileria* spp. including *T. lestoquardi*, *T. luwenshuni* and *T. uilenbergi* [1, 2]. In addition, there is frequent infection of small ruminants with non-pathogenic *Theileria* spp., including *T. ovis*, *T. separata*, and *T. recondite* [1, 2]. In China, *T. uilenbergi* and *T. luwenshuni* are highly pathogenic, especially in sheep, and cause severe clinical signs [3]. *Theileria lestoquardi* is among the most common and pathogenic *Theileria* spp. of small ruminants in the Arabian Peninsula and is highly prevalent in the Gulf Cooperation Council (GCC) countries, causing high morbidity and mortality among indigenous sheep [4]. Recovery from acute infection following chemotherapy is often followed by persistence of some parasites—referred to as the carrier state—which is often undetectable microscopically but enables transmission by the tick vector [5, 6]. The carrier state creates a significant risk for the spread of disease into other theileriosis-free areas where the vector is present. Chronic asymptomatic carriage of *Theileria* spp. has been linked to significant losses in livestock productivity [7].

Previous surveys in Oman have demonstrated high rates of asymptomatic infection with *Theileria* spp. among cattle (72.3%) and sheep (37.5%) [8]. Interestingly, sheep frequently had mixed infections with *T. lestoquardi* (pathogenic) and *T. ovis* (non-pathogenic) [5, 9], which are both transmitted by *Hyalomma anatolicum*, the predominant tick species identified on animals examined in Oman [5]. Our most recent data illustrated a potential competitive interaction between these two *Theileria* spp.; a substantial reduction in the risk of mortality due to *T. lestoquardi* was observed in mixed-parasite infections, suggesting that *T. ovis* confers protection against the pathogenic effect of *T. lestoquardi* infection [10]. A protective effect of mixed-species infection (*T. lestoquardi* + *T. ovis*) against severe malignant ovine theileriosis (MOT), is in line with a large body of data on apicomplexan parasites indicating that mixed infections with different species may modulate the virulence and pathogenicity of each other [11]. Evidence of a cross-species protective effect has been shown in a number of apicomplexan parasites, such as the reciprocal effect between the bacterium *Anaplasma phagocytophilum* and *Babesia microti* parasite in field voles [12], and the suppression of *B. divergens* by *A. phagocytophilum* in cattle [13]. More recently, a field epidemiological study in indigenous zebu calves in East Africa demonstrated an 89% reduction in mortality due to *T. parva* infection (East Coast fever) in the presence of less pathogenic *Theileria* spp. (*T. mutans* and *T. velifera*) [14]. Understanding the consequences of mixed infections to variations in *Theileria* spp. pathogenicity is critical for designing control measures aimed at reducing morbidity and mortality.

Infections with *Theileria* parasites induce a range of hematologic and biochemical changes associated with the intra-leukocyte schizont and intra-erythrocyte piroplasm stages. The severity of these changes is related to the virulence of the strain, the infectious dose, the animal breed, and immune status [15–17]. Experimental infections of cattle and sheep, respectively, with *T. annulata* and *T. lestoquardi*, have demonstrated a significant progressive decrease in the concentration of hemoglobin, hematocrit, and red blood cells, as well as leukopenia and leukocytolysis [18–20]. The absence of hemoglobinemia indicates that the decrease in hematocrit and red blood cells may be due to erythrocyte destruction by mononuclear phagocyte system rather than intravascular lysis [17, 20].

The present study extends our previous findings in Oman by examining clinical and hematological indices in sheep naturally co-infected with *T. lestoquardi* and *T. ovis* to determine whether interactions between the pathogenic and less pathogenic species of *Theileria* significantly reduces the impact of theileriosis. Such information can indicate whether infection of the non-pathogenic *T. ovis* can be utilized as a predictor of clinical outcome of MOT. In addition, we examined the influence of genetic complexity on disease indices and compared the extent of diversity of *T. lestoquardi* in the current study to that collected in other regions in 2014, to investigate temporal changes in parasite structure in Oman.

## Methods

### Study area and sampling

**Fig. 1:**
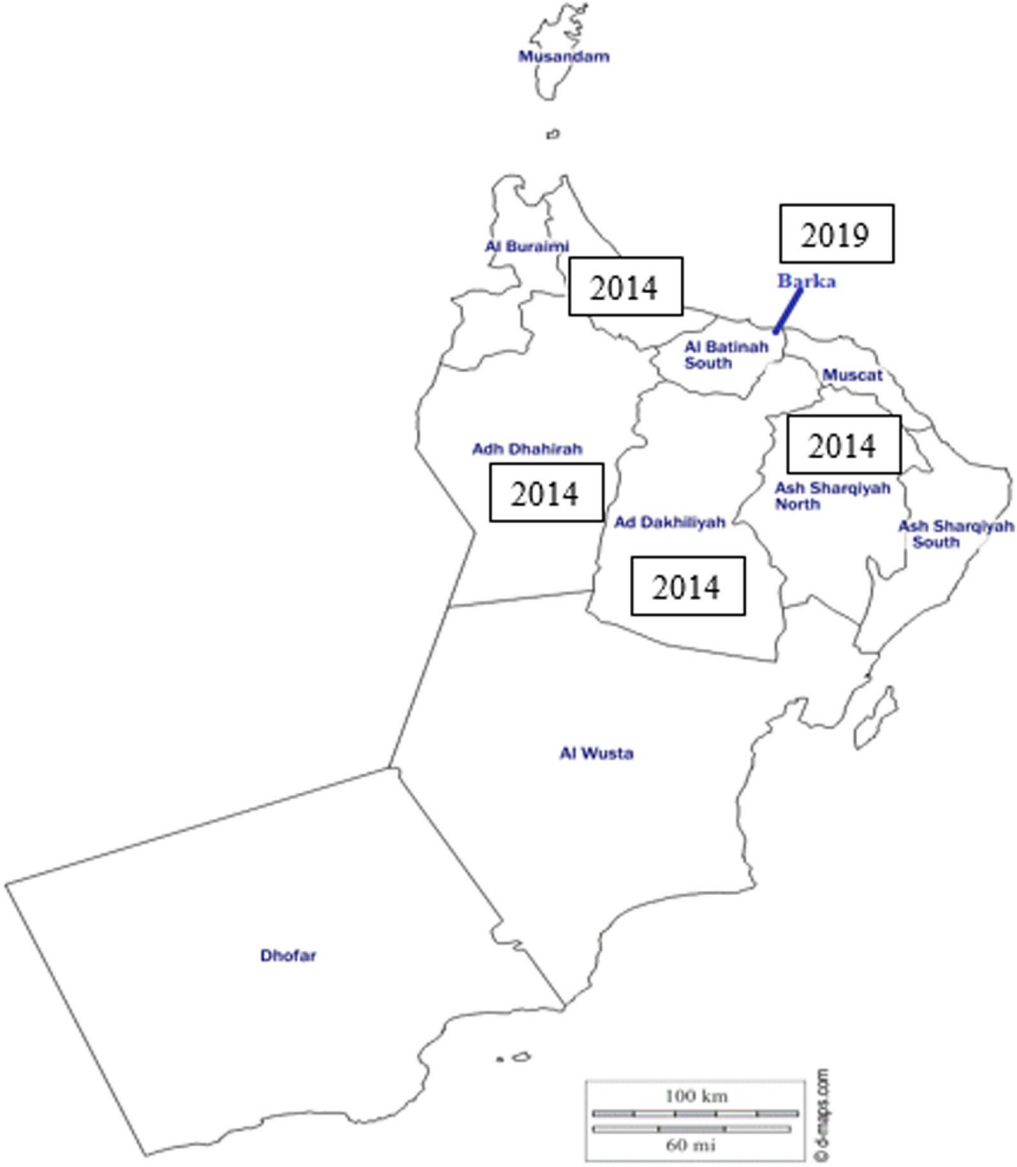
Locations of collection sites from Barka, Al-Bāţinah South Governorate (2019) and other regions in Oman (2014), Al-Bāţinah, Adh Dhahirah, Ad Dakhiliya, As Sharqoyah, Dhofar. 2014 and 2019 refer to study years when samples were collected

A cross-sectional study was conducted in Barka (Al-Bāţinah South Governorate, Fig. 1) between July and November 2019. A total of 390 blood samples were collected from 16 sheep farms, number of examined animals per farm ranged between 10 to 56. The examined animals include indigenous breed, North of Oman breed, and exotic, imported from Saudi Arabia, Somalia, India, and Iran. Some of the screened farms had a history of theileriosis. Lymph node palpation, rectal temperature, and the color of the mucus membranes of the eyes were assessed during sample collection. In addition, the age, sex, and breed of the animals were recorded. The study area is characterized by a hot climate with a long summer season from mid of April to October. The temperature can reach up to 50.8 °C. Characteristically, there is only a small amount of rainfall throughout the year with 100 mm average annual rainfall, mostly in January. Animals are kept outdoors, in secured enclosures, due to a lack of open natural pastures, and fed on Rhodes grass hay. They are herded into shelters; however, movement from farm to farm is not an uncommon practice.

The research was performed in accordance with the relevant guidelines and regulations of the ethical and animal welfare code of Sultan Qaboos University, Oman and the Ministry of Agriculture and Fisheries, Oman. Oral consent was obtained from the farm owner before drawing blood from animals.

### Hematological assessment

Five milliliters of blood was collected from individual animals via venipuncture into Vacutainer tubes containing EDTA and immediately transported to the laboratory in iceboxes for determination of hematological parameters and DNA extraction.

Hematological parameters including total erythrocyte count (RBC), hematocrit (HCT), hemoglobin concentration (Hb), mean corpuscular volume (MCV), mean corpuscular hemoglobin concentration (MCHC), and total white blood cell count (WBC), as well as differentials, were determined using an Exigo H400 veterinary hematology analyzer (Spånga, Sweden).

### Detection and quantification of *T. lestoquardi *and *T. ovis*

DNA was extracted from 200 µl of blood, using the QIAamp DNA mini kit (Qiagen, Germany) according to the manufacture’s instruction, and stored at −20 °C. *Theileria* spp. were identified by sequencing polymerase chain reaction (PCR) products obtained using pan-*Theileria* primers, targeting the *18S rRNA* as described elsewhere [21].

The density of *T. lestoquardi* and *T. ovis* parasites was quantified using real-time polymerase chain reaction (qPCR) to determine the gene copy number. Species-specific primers and probes targeting the *18S rRNA* gene of *T. lestoquardi* and *T. ovis* were used; details of assay optimization are described elsewhere [10]. Standard curves were generated using quantified DNA of *T. lestoquardi* (9.26 × 10^10^ *18S rRNA* gene copies/μL) and *T. ovis* (5.3 × 10^10^ *18S rRNA* gene copies/μL).

### Typing of *T. lestoquardi* microsatellites

*T. lestoquardi* was genotyped using a panel of ten unlinked polymorphic microsatellite DNA markers as described elsewhere [9]. Labeled PCR products were mixed with Gene-Scan™ 500 ROX internal size standard (Applied Biosystems, UK) for capillary electrophoresis on an ABI 3130XL Genetic Analyzer (Applied Biosystems, UK). GeneMapper software version 4 (Applied Biosystems, UK) was used for scoring allele sizes and quantifying electropherogram peak heights for samples containing multiple alleles per locus. Multiple alleles per locus were scored if electrophoretic peaks corresponding to minor alleles were > 32% of the height of the predominant allele [22].

### Data analysis

Data as mean and standard deviation for hematological parameters were compared among the fixed factors (sheep infected with *T. lestoquardi* alone, mixed infection “*T. lestoquardi* and *T. ovis*”, and uninfected group). For this purpose, multivariate analysis of covariance by controlling for potential confounding factors such as age, sex, breed, and farm was performed. Hematological parameters, including WBCs, differential WBCs, and platelets, were log-transformed to meet the assumption of normality. Categorical responses were expressed as a percentage, and the association of potential risk factors such as sex, age, breed, presence of ticks, enlargement of lymph nodes, and pallor of ocular mucus membrane were tested individually using Pearson’s chi-squared or Fisher’s exact test using univariate logistic regression (Table 1). The model of binary logistic regression was performed using the backward stepwise method to test the significance of the variables tested by the univariate analysis (Table 2). Odds ratio and respective 95% confidence interval (CI) were also calculated for the significant variables. Odds ratio with significant result and value less than one were inversed to facilitate interpretation. The effect of parasite density on hematology indices was assessed using regression method. All analyses were done with SPSS Statistical Software for Windows, version 24.0 (IBM Corp., Armonk, NY, USA). All tests were two-tailed, and *P*-values of less than 0.05 were considered statistically significant.

**Table 1:** Univariate analysis for the association of potential risk factors with *Theileria* sp. infection among sheep in Barka, Oman

Variables	Number (%)	PCR		Chi-square test	*P*-value
		Positive *n* = 75 (19.2%)	Negative *n* = 315 (80.8%)		
Sex					
Male	64 (16.4)	18 (28.1)	46 (71.9)	3.90	0.05
Female	326 (83.6)	57 (17.5)	269 (82.5)		
Age					
Less than 1 year	122 (31.3)	19 (15.6)	103 (84.4)	3.02	0.22
1–2 years	106 (27.2)	26 (24.5)	80 (75.5)		
More than 2 years	162 (41.5)	30 (18.5)	132 (81.5)		
Breed^a^					
Exotic	75 (19.2)	27 (36)	48 (64)	18.86	0.001
Cross-bred	24 (6.2)	1 (4.2)	23 (95.8)		
Indigenous	291( 64.6)	47 (16.2)	244 (83.8)		
Presence of ticks					
Yes	178 (45.6)	37 (20.8)	141 (79.2)	0.51	0.48
No	212 (54.4)	38 (17.9)	174 (82.1)		
Lymph node					
Normal	135 (34.6)	30 (22.2)	105 (77.8)	1.19	0.28
Enlarged	255 (65.4)	45 (17.6)	210 (82.4)		
Mucous membrane color					
Normal	334 (85.6)	63 (18.9)	271 (81.1)	0.20	0.65
Pale	56 (14.4)	12 (21.4)	44 (78.6)		

^a^Significant difference

**Table 2:** Model of binary logistic regression using backward stepwise method of the hypothesized risk factors with the frequency of *Theileria* sp. infection

Variables	*β*	SE *β*	*P*-value	Adjusted odds ratio	95% CI	
					Lower	Upper
Breed						
Exotic	0.00	–	–	1	–	–
Cross-bred	−2.52	1.05	0.016	0.08	0.01	0.63
Indigenous	−1.05	0.30	0.001	0.35	0.19	0.62

*β*: logistic coefficients. SE: standard error, CI: confidence interval. Odds ratio of sheep breed: exotic sheep were 2.9 (CI 1.66–5.14) and 12.9 (CI 1.65–101.19) times as likely to be *Theileria*-positive as indigenous and cross-bred sheep, respectively

Microsatellite allele data were filtered to retain only minor alleles having a peak height of > 32% of the corresponding predominant alleles if more than one allele was present at any given locus. Genetic diversity metrics were calculated for the entire dataset using GenAlEx 6.5 [23]. Expected heterozygosity was calculated using the formula for “unbiased heterozygosity,” also termed haploid genetic diversity, *He* = [*n*/(*n* − 1)][1 − ∑*p*2], where *n* is the number of isolates and *p* the frequency of each different allele at a given locus [24]. Population differentiation was assessed by estimating Wright’s FST index using the FSTAT computer program version 2.9.3.2. Two estimators of FST (G′ST and θ) [25] were used to estimate genetic differentiation between imported parasites from the Indian subcontinent and Africa.

Multiplicity of infection (MOI), defined as the presence of multiple genotypes per infection, was determined by the detection of more than one allele at a given locus. To avoid over-estimation of low-abundance alleles, only minor alleles having a peak height of > 33% (one third) of the corresponding predominant alleles were accepted. The proportion of samples with more than one allele across ten loci was used to represent MOI.

Variance of mismatch values (VD) were compared to values of L (the upper confidence limits of Monte Carlo simulation and parametric tests), and where VD > L linkage disequilibrium is assumed [26].

## Results

### Demographic data

A total of 390 sheep, comprising three breeds, indigenous (*n* = 291), exotic (*n* = 75), and crossbreeds (*n* = 24), were screened for the presence of *T. lestoquardi* by PCR assay of *18S rRNA* (Table 1). The majority of sheep 212 (54.4%) were infested with adult ticks, and 57 (14.6%) had elevated rectal temperatures > 40 °C. Enlargement of lymph nodes was observed in 255 sheep (65.4%), either unilaterally (32.6%, *n* = 127) or bilaterally (32.8%, *n* = 128). In addition, pale mucus membrane was recorded in 56 animals (14.4%).

*Theileria* spp. were detected in 75 (19.2%) of the examined animals; 8 (10.6%) carried single *T. lestoquardi,* 2 (2.7%) had single *T. ovis*, and 65 (86.7%) harbored mixed-species infections with *T. lestoquardi* and *T. ovis*. There was a great variation in the prevalence of *Theileria* spp., in the examined farms, ranging between 5.9% and 53.5%.

### Clinical and hematological indices among sheep with different *Theileria* species

*Theileria* spp. prevalence was not associated with age (*p* = 0.22) or sex (*p* = 0.05) of the animals across the region. Similarly, no differences were observed between sheep infested with ticks and tick-free sheep (*p* = 0.48) (Table 1) as well as animals with enlarged or non-enlarged lymph nodes (*p* = 0.28). However, the infection rate was significantly higher among exotic breeds (36%, 27/75) (odds ratio 12.9, CI 1.65–101.19) compared to indigenous (16.2%, 47/291) and crossbreeds (4.2%, 1/24) (odds ratio 2.9 CI 1.66–5.14) (Table 2).

Hematological indices including hematocrit, RBC counts, Hb, MCV, MCHC, WBC, eosinophils, and lymphocytes were not correlated with detection of *Theileria* infection. Nonetheless, compared to animals with mixed infections (*T. lestoquardi* plus *T. ovis*), sheep with single *T. lestoquardi* infections showed a lower average, but not significant values, of total WBCs (8.49 ± 2.5 vs. 9.76 ± 2.74, *p* > 0.05), lymphocytes (3.86 ± 1.17 vs. 4.34 ± 1.41), monocytes (0.70 ± 0.17 vs. 0.79 ± 0.25), eosinophils (1.03 ± 0.58 vs. 1.28 ± 0.78), and neutrophils (2.94 ± 1.02 vs. 3.25 ± 1.31, *p* > 0.05). However, all counts were within our normal clinical reference ranges (Table 3).

**Table 3:** Multivariate analysis of covariance for hematological indices among sheep infected with *T. lestoquardi* alone, mixed infection (*T. lestoquardi* and *T. ovis*), and uninfected groups

Blood indices	Reference range	Groups Mean ± SD			*F* test	*P*-value
		*T lestoquardi* *n* = 8 (2.1%)	Mixed infection *n* = 65 (16.8%)	Uninfected *n* = 315 (81.2%)		
RBC	(9–15) × 10^6^ /μL	10.42 ± 1.59	9.71 ± 1.31	9.96 ± 1.61	0.710	0.494
HCT	(27–45) %	31.44 ± 3.87	29.74 ± 4.23	29.74 ± 4.43	0.231	0.794
Hb	(9–15) g/dL	10.80 ± 1.38	10.19 ± 1.40	10.27 ± 1.53	0.255	0.775
WBC	(4–8) × 10^3^μL	8.49 ± 2.50	9.76 ± 2.74	9.97 ± 2.81	1.845	0.159
Lymphocyte	(2–9) × 10^3^μL	3.86 ± 1.17	4.34 ± 1.41	4.32 ± 1.22	2.134	0.120
Monocytes	(0–0.75) × 10^3^μL	0.70 ± 0.17	0.79 ± 0.25	0.79 ± 0.24	1.246	0.289
Neutrophils	(0.7–6.0) × 10^3^μL	2.94 ± 1.02	3.25 ± 1.31	3.42 ± 1.47	0.458	0.633
Eosinophils	(0–1.0) × 10^3^μL	1.03 ± 0.58	1.28 ± 0.78	1.29 ± 0.75	0.205	0.814
Platelets	(800–1100) × 10^3^μL	386.05 ± 175.23	333 ± 141.08	359.03 ± 144.61	0.555	0.575

*Only two sheep harbored single *T. ovis* infection

### Parasite density and clinical and hematological indices

Total *T. lestoquardi* density among infected sheep, as judged by results of the PCR assay on blood samples, varied widely, between 1.03 and 5.99 log_10_ *18S rRNA* copies/μL. The estimated mean density in single *T. lestoquardi* infection was higher (4.77 log_10_ *18S rRNA* copies/μL blood, 95% CI 4.5 – 5.1 log_10_) than mixed infection (4.2 log_10_ *18S rRNA* copies/μL blood, 95% CI 3.9–3.5 log_10_), but this difference was not significant (*p* = 0.12). Similarly, *T. ovis* density among the infected sheep varied widely, between 1.06 and 5.30 log10 *18S rRNA* copies/μL (mean = 4.03). Single *T. ovis*, detected in only two samples, had a higher average density of 4.3 log_10_ *18S rRNA* copies/μL blood (95% CI 3.21–5.37 log_10_), than the mean density of *T. ovis* among mixed infection (4.02, log_10_ *18S rRNA* copies/μL blood, 95% CI 3.84–4.22 log_10_).

In mixed infections, *T. lestoquardi* was present at a slightly higher density (4.2 log_10_ *18S rRNA* copies/μL blood, 95% CI 3.9–4.5 log_10_) than *T. ovis* (4.02 log_10_ *18S rRNA* copies/μL blood, 95% CI 3.8–4.2 log_10_) (*p* = 0.184). Nonetheless, there was a highly significant association between the densities of the two species in mixed infection in individual animals (*p* < 0.0001).

Density of both *T. lestoquardi* and *T. ovis* was not associated with gender (*p* > 0.25), breed (*p* > 0.47), presence of ticks (*p* > 0.25) or enlargement of lymph nodes (*p* > 0.45). Moreover, *T. lestoquardi*, when present as a single infection, had no significant effect on any of the examined hematological indices (*p* > 0.05).

### Genetic diversity and structure of *T. lestoquardi*

Seventy-three *T. lestoquardi* isolates from Barka were successfully genotyped for ten species-specific microsatellites. Eight carried *T. lestoquardi* but not *T. ovis* and 65 harbored mixed species (*T. lestoquardi* plus *T. ovis*).

The total number of alleles detected for each of the *T. lestoquardi* loci ranged from 5 for TL_MS25 *to* 22 for TL_MS280, and the number of alleles per locus within a single sample ranged from 1 to 6. A high level of polymorphism was observed among six loci (TL_MS205, TL_MS281, TL_MS280, TL_MS07, TL_MS13, and TL_MS19) while a moderate level of diversity was observed for a further 4 loci (TL_MS04, TL_MS16, TL_MS18, and TL_MS25) (Table 4). The average heterozygosity among single *T. lestoquardi* infections was significantly lower (*H*_*e*_ = 0.685), ranging from 0.00 to 0.893, than that in mixed (*T. lestoquardi/T. ovis*) infections (*H*_*e*_ = 0.76), ranging from 0.590 to 0.933 (*p* = 0.0001).

**Table 4:** Allelic diversity and unbiased heterozygosity (*He*) at 10 micro- and mini-satellite loci among 73 T*. lestoquardi* isolates collected in Barka in 2019 and 190 isolates examined in 2014 in different regions in Oman

Population	TL_ MS05	TL_ MS281	TL_ MS04	TL_ MS07	TL_ MS13	TL_ MS16	TL_ MS19	TL_ MS280	TL_ MS25	TL_ MS18	Average
2019 (*n* = 73)	0.861	0.737	0.673	0.857	0.925	0.594	0.831	0.893	0.579	0.662	0.772
2014 (*n* = 190)	0.867	0.839	0.359	0.705	0.796	0.441	0.665	0.760	0.121	0.548	0.582

### Multiplicity of infection

Seventy-two (98.7%) of the above 73 *T. lestoquardi* samples carried multiple parasite genotypes. The high prevalence of genotype multiplicity in Barka (98.7%) is consistent with that seen in other sites in 2014 (96%) in Oman. The extent of multiplicity and the number of genotypes per sample were slightly lower among single *T. lestoquardi* infection, ranging between 2 and 5, mean 3.4 (SD ± 0.92), compared to mixed (*T. lestoquardi*/*T. ovis*) infection, ranging between 1 to 6, mean 3.59 (SD ± 1.04).

### Linkage disequilibrium

LD was estimated using the standard index of association to investigate whether the high diversity observed in *T. lestoquardi* in Barka could be explained by a panmictic population structure and high rates of recombination in the tick vector. When all samples collected in all farms were treated as a single population, an I^S^_A_ value of (0.027) and a V_D_ value (2.06) greater than L (1.97) was obtained, indicating LD.

### Population sub-structuring

F_ST_ values (G′_ST_ and θ) were estimated to measure the level of genetic relatedness of *T. lestoquardi* in Barka in 2019 compared to a previous dataset from parasites isolated in different sites in Oman in 2014 employing the same panel of markers [9].

**Fig. 2:**
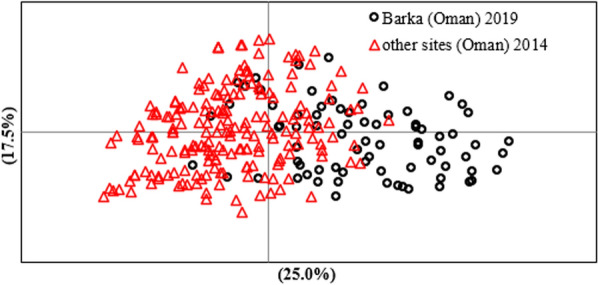
Principal coordinate analysis (PCoA) of *T. lestoquardi populations* from Barka and other regions in Oman collected on 2014. PCoA was performed on the multi-locus genotype data representing each of the populations sampled. The proportion of the variation in the dataset explained by each axis is indicated in parentheses

No differentiation was observed between the *T. lestoquardi* population samples collected in different sites in Oman in 2014 (*G*′_ST_ = 0.024 and *θ* = 0.024). However, a moderate level of differentiation (*G*′_ST_ = 0.096 and *θ* = 0.04) was observed between parasite populations in Oman in 2014 and Barka in 2019. The estimated differentiation is supported by principal coordinate analysis (PCoA) (Fig. 2). PCoA demonstrated evidence of structuring, with some haplotypes distributed independently of year of collection. Haplotypes from 2014 and 2019 are overlapped, however, a few lineages from 2019 diverge from the rest of individuals.

## Discussion

The present study provides additional evidence for within-host interaction of *T. ovis* and *T. lestoquardi* in small ruminants in Oman [10]. The density of both parasites was higher when present as a single species infection (presence of one species alone), compared to mixed-species infections (presence of the two species), which we believe provides support for the concept of heterologous protection, the ability to limit parasite load conferred by mixed infection with both parasites [14]. This was partly reflected as a disproportionately higher prevalence of mixed infection (*T. lestoquardi* and *T. ovis*) among animals, compared to single infection with each, supporting the hypothesis of heterologous protection against disease in endemic areas [14]. This is consistent with epidemiological surveys in a different region in Oman, where mixed infection with *T. lestoquardi* and *T. ovis* in sheep was remarkably higher than single infection of each species separately [5]. However, there is information on the extent of mixed-species infection in the main tick vector in the region*, Hyalomma anatolicum.*

Within-host interaction between *T. ovis* and *T. lestoquardi* is a reasonable explanation for the occurrence of *T. lestoquardi* (pathogenic parasite) at a lower density when present as a mixed infection (4.2 log_10_ *18S rRNA* copies/μL blood, 95% CI 3.9–3.5 log_10_) compared to single infection (4.77 log_10_ *18S rRNA* copies/μL blood, 95% CI 4.5–5.1 log_10_), though this difference was not significant (*p* = 0.12). Furthermore, the pathogenic parasite (*T. lestoquardi*) was present at a relatively higher density (4.2 log_10_ *18S rRNA* copies/μL blood, 95% CI 3.9–4.5 log_10_) than the non-pathogenic (*T. ovis*) (4.02 log_10_ *18S rRNA* copies/μL blood, 95% CI 3.8–4.2 log_10_) (*p* = 0.184). Unfortunately, few animals displayed single *T. ovis* infection (*n* = 2). However, in those that showed single *T. ovis*, the parasitemia was always relatively higher compared to mixed infections (4.3 vs. 4.02 log_10_ *18S rRNA* copies/μL). The lower *T. lestoquardi* parasitemia in mixed infection is indicative of immune pressure (potentially innate), which is expected to impact both parasites (*T. lestoquardi* and *T. ovis*). The above findings are in line with our most recent data in a cohort study of indigenous sheep in Oman, which revealed a significantly higher *T. lestoquardi* density in single infection versus mixed infection [10].

The observation of predominantly mixed-species infection (86.7%) among the asymptomatic animals examined in the present study is in line with the hypothesis of heterologous protection against disease in endemic areas [14]. Mixed-species infection of some apicomplexan parasites, as well as co-existence of multiple genotypes of the same species, have been considered advantageous to the host, often associated with reduced morbidity and mortality [27]. Evidence for a protective effect of mixed *Theileria* spp., in mortality of East Coast fever due to *T. parva* infection, in the presence of less pathogenic *Theileria* spp. (*T. mutans* and *T. velifera*) has been demonstrated in indigenous African *Bos indicus* calves [14]. This is consistent with our most recent work in Oman that linked mixed infection (*T. lestoquardi* plus *T. ovis*) to significant reduction of mortality among local breeds of sheep [10]. The present study extended these earlier findings and examined clinical and hematological indices in sheep carrying mixed (*T. lestoquardi* plus *T. ovis*) compared to single *T. lestoquardi* infections. Lower levels of WBCs, lymphocytes, and neutrophils were seen among single *T. lestoquardi* infection compared to mixed infection, however, these differences were not associated with overt clinical signs and were not statistically significant (Table 3). The limited proportion of animals with single *T. lestoquardi* infection (10.6%) in the present study and related potential variation caused by other pathogens in hematological indices precluded statistical validation of the impact of type of infection on some morbidity markers. Thus, further studies to substantiate these findings would require data from a larger sample of animals infected with single infections (*T. lestoquardi* and *T. ovis*) to obtain statistically valid comparisons.

A high level of genetic diversity and genotype multiplicity of *T. lestoquardi* was detected in infected sheep in Barka. However, no differences in the extent of diversity were seen among parasites causing single (*He* = 0.68) versus mixed infection (*He* = 0.76). Similarly, multiple genotypes were common among single and mixed *T. lestoquardi* infections with equal extent of multiplicity suggesting a common mechanism for regulation of the dynamics of genotypes. The matched level of *T. lestoquardi* diversity detected in animals with single and mixed-species infection suggests that both forms of infection are equally susceptible to establishment of multiple genotype populations. This can be attributed to high abundance and infestation of *H. anatolicum*, which can promote cross-mating and recombination within ticks to generate novel parasite genotypes for infection of sheep. The above is consistent with the high genetic diversity and multiplicity of infection reported for *T. lestoquardi* populations in Oman [9] and Sudan [28].

Very low levels of genetic differentiation were detected between *T. lestoquardi* parasites collected in 2014 in different sites in Oman, including Batinah where the current study was carried out, with an average pairwise *F*_ST_ value of 0.024. This is consistent with the high rate of tick infestation among the infected sheep, which can lead to a high level of cross-mating and recombination in ticks, to generate novel genotypes. However, a moderate level of genetic differentiation was measured between *T. lestoquardi* parasites in Barka examined in the current study in 2019 and parasites in other sites in Oman, with *F*_ST_ values of 0.048, though, complete separation between the parasite populations was not observed (Fig. 2). This moderate level of differentiation is consistent with the data from similar analysis between *T. annulata* in Tunisia and Turkey (*F*_ST_ values of 0.049) [29]. A more profound genetic differentiation has been observed between *T. annulata* and *T. lestoquardi* in Oman and populations in widely separated countries, consistent with geographical and trade barriers hindering gene flow [9, 21, 28]. For example, a high level of differentiation (*F*_ST_ = 0.295) was evident between *T. lestoquardi* populations in Oman and Sudan, while no temporal differentiation was observed between *T. lestoquardi* population collected in Sudan in 2013 compared to 2016 (*F*_ST_ = 0.29) [28]. Unlike the findings in Sudan, the present study revealed temporal moderate differentiation between parasites in Barka (2019) and other sites in Oman (2014), which is likely to be due to trade and importation of asymptomatic parasite carriers from outside the country through commercial trade of sheep, allowing admixture and intercrossing with local parasite populations in Oman. This is consistent with observations of similar patterns of admixed genetic structure of populations of livestock in Oman, cattle and goats, with African and Near East origin [30, 31], facilitating dissemination of vector-borne parasites via trade [32]. A large proportion of the sheep examined (19.2%) in the present study were of exotic breed, which represents a large proportion of imported animals (Table 1) that frequently are carriers of *T*. *lestoquardi*. In March 2020, Oman imported 138,200 head of sheep, and 8427 head of cattle from different parts of the world, including Australia, Sudan, South Africa, and Somalia [33]. Animal movement between countries poses a significant threat for dissemination of parasites and their vectors. In turn, this can lead to a high level of gene flow between parasite populations from close geographical locations.

In summary, the present study provides additional evidence of within-host interaction between the *T. lestoquardi* and *T. ovis*, and noticeable differences in some hematological indices and parasite density among single *T. lestoquardi* infection compared to mixed infection. The extent of genetic diversity of *T. lestoquardi* that exists as single infection is similar to that in mixed infection suggesting that the parasites in the two groups are homogenous. The *T. lestoquardi* genotypes detected in Barka in 2019 showed moderate differentiation compared to those seen in other sites in Oman in 2014, suggesting that new parasite strains may have been introduced in the area via importation of infected animals.

## Data Availability

All data generated or analyzed during this study are included in this published article.

## Abbreviations

GCC: Gulf Cooperation Council
MOT: Malignant ovine theileriosis
RBC: Red blood cells
HCT: Hematocrit
Hb: Hemoglobin
MCV: Mean corpuscular volume
MCHC: Mean corpuscular hemoglobin concentration
WBC: Total white blood cell count
PCR: Polymerase chain reaction
qPCR: Quantitative polymerase chain reaction
MOI: Multiplicity of infection
VD: Variance of mismatch values
LD: Linkage disequilibrium
PCoA: Principal coordinate analysis
*He*: Heterozygosity
I^S^_A_: Association of index
F_ST_: Wright’s fixation index
